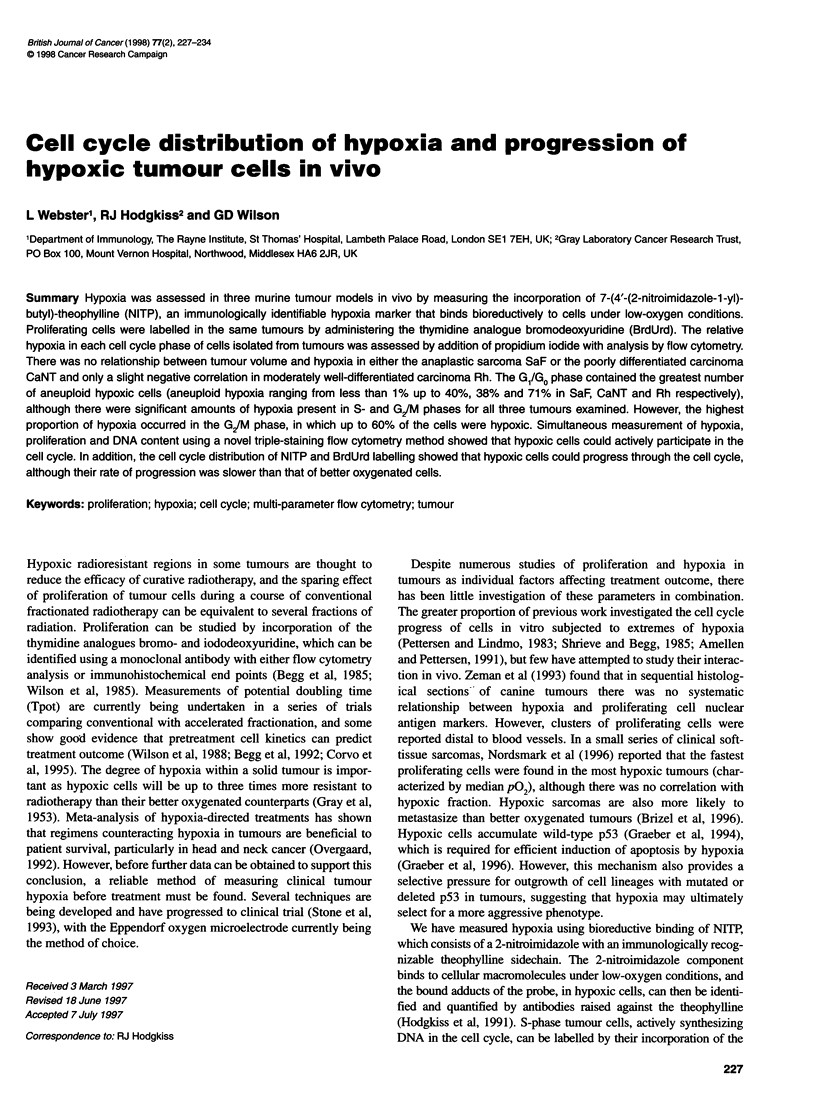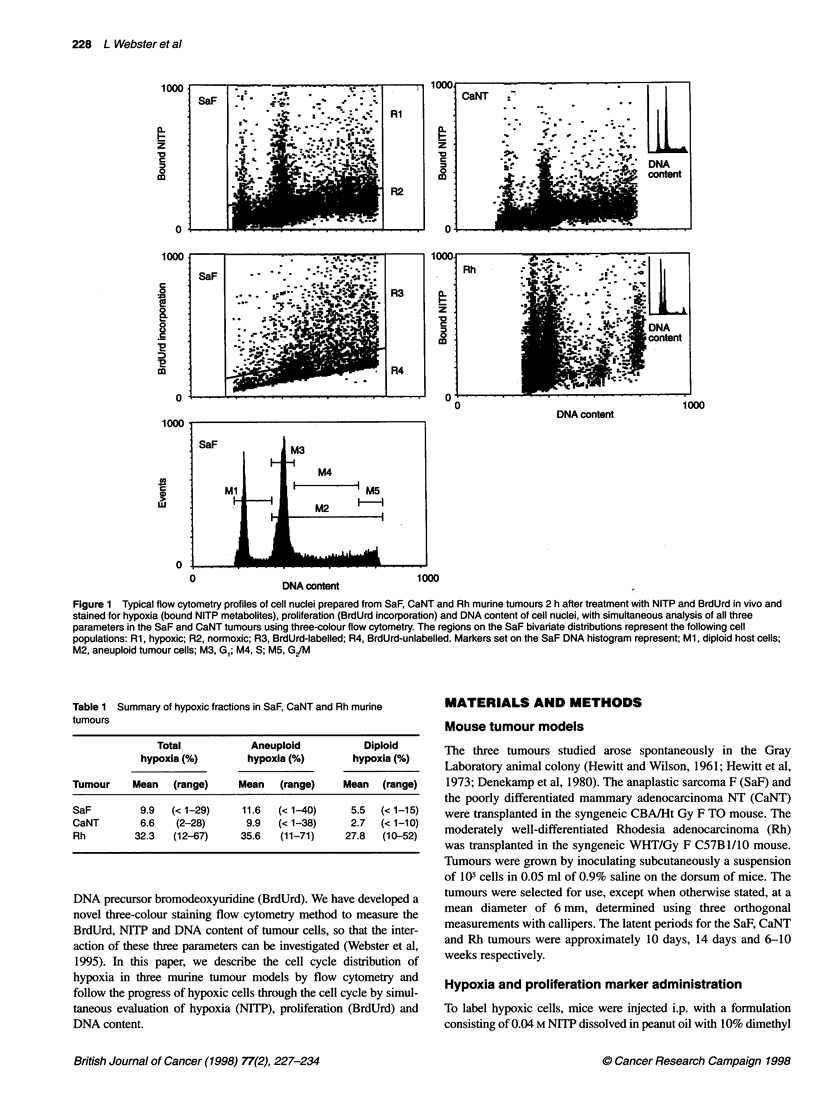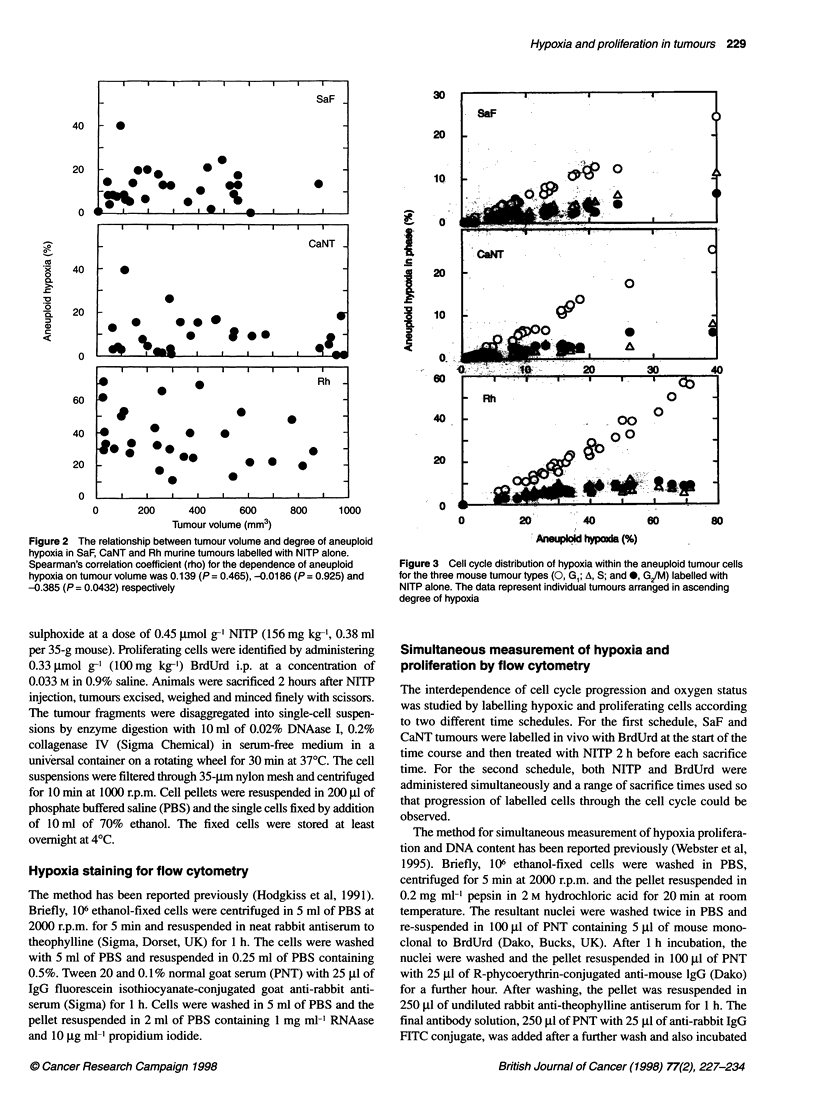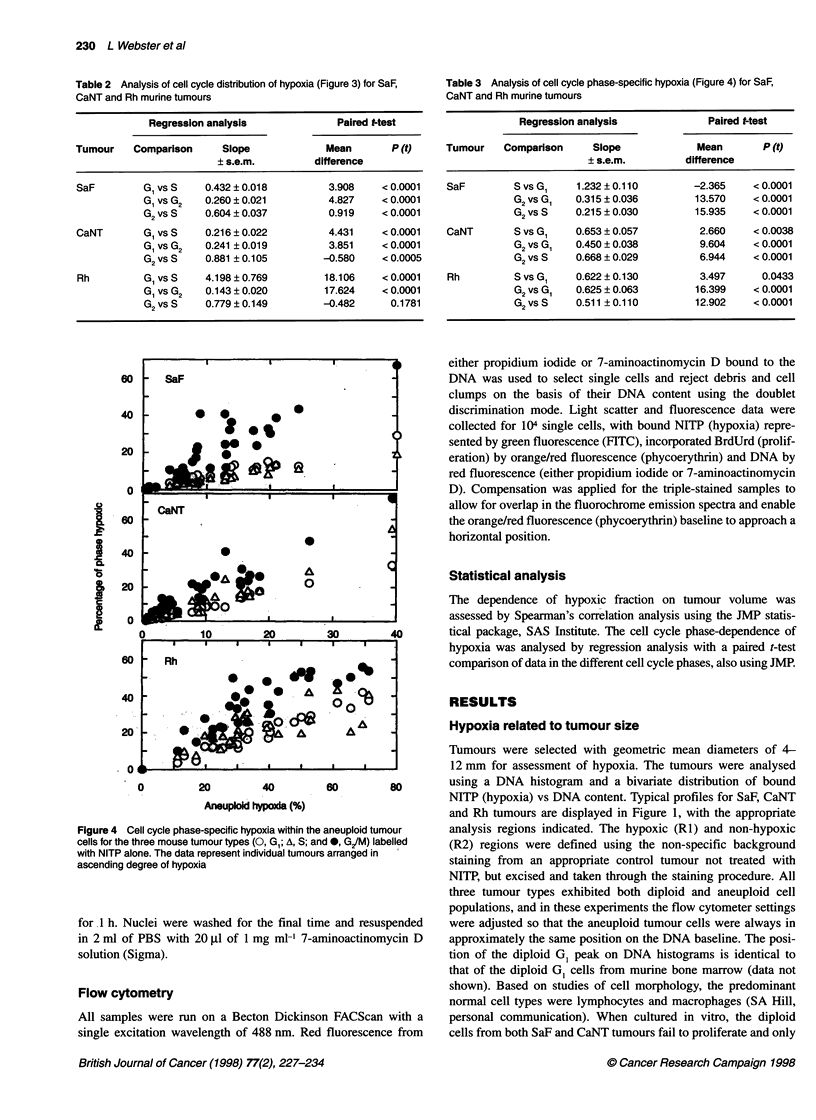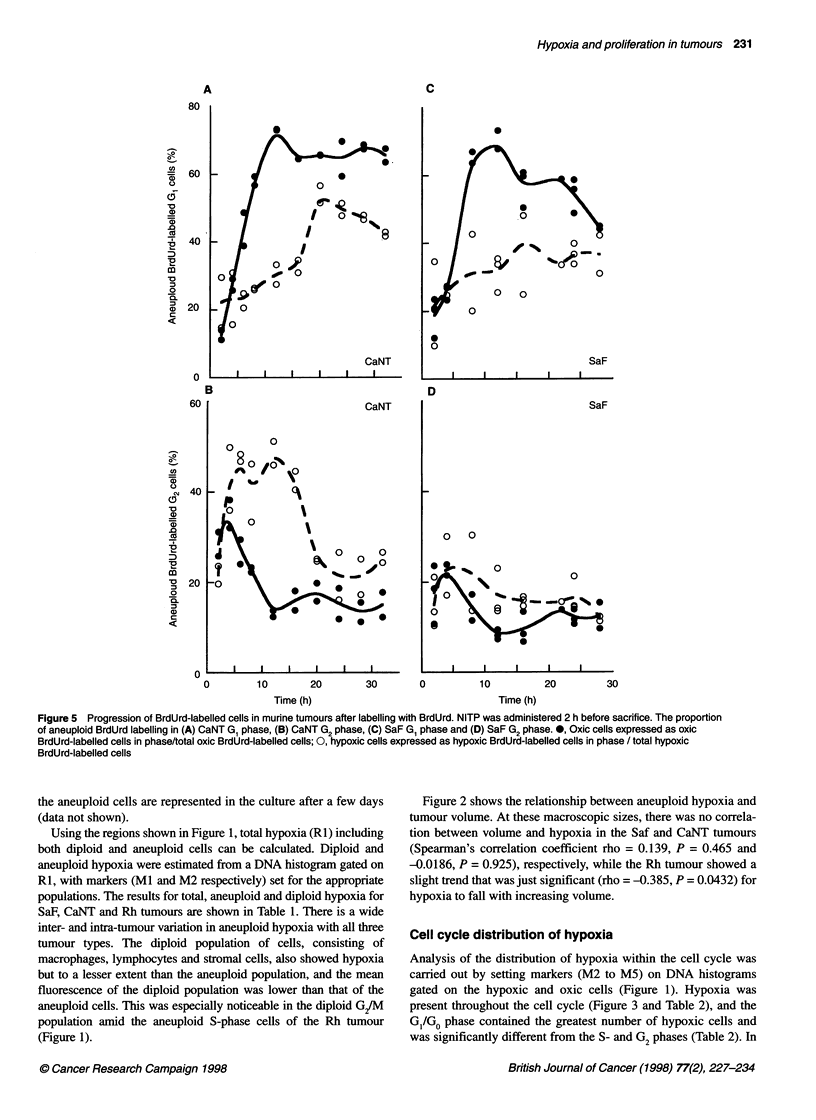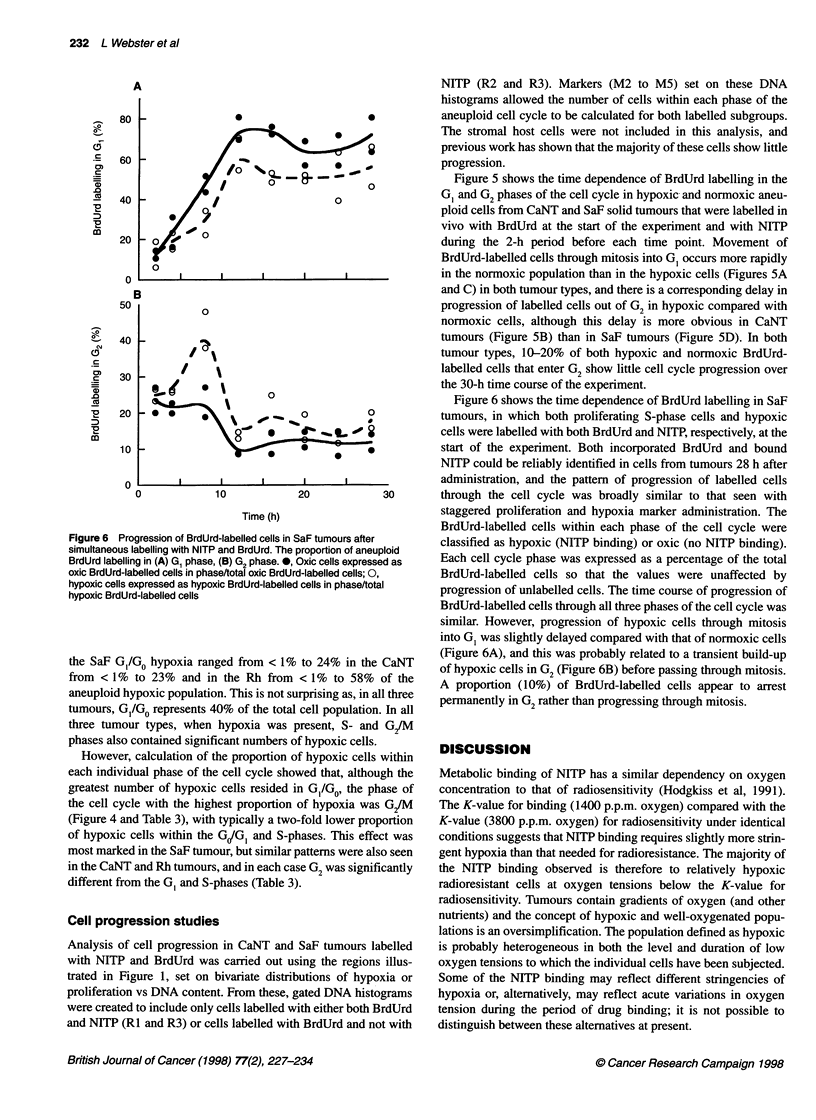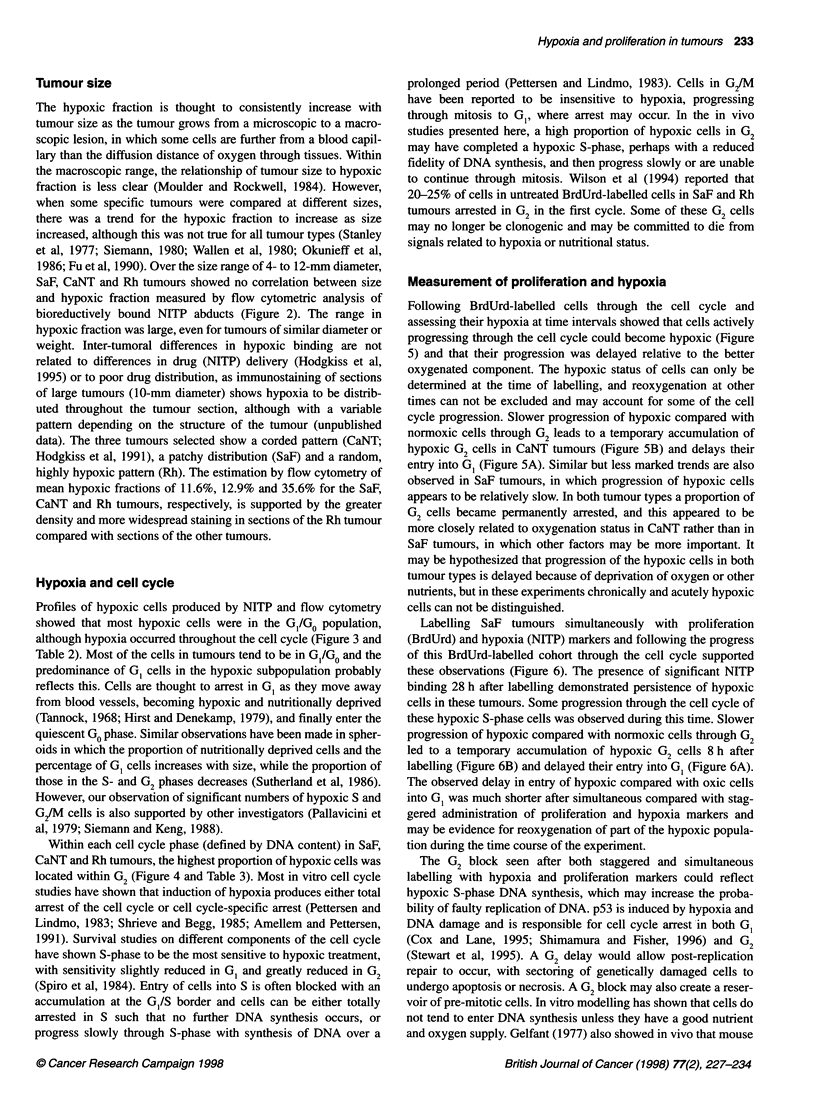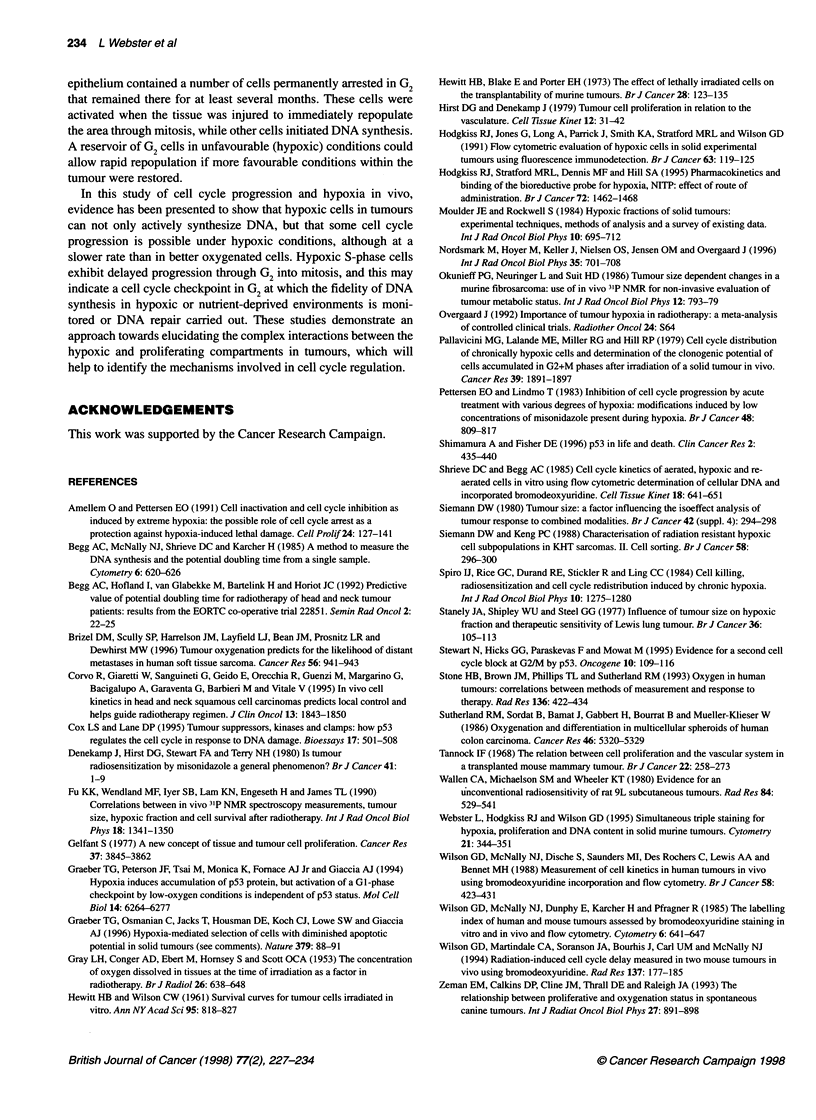# Cell cycle distribution of hypoxia and progression of hypoxic tumour cells in vivo.

**DOI:** 10.1038/bjc.1998.38

**Published:** 1998

**Authors:** L. Webster, R. J. Hodgkiss, G. D. Wilson

**Affiliations:** Department of Immunology, The Rayne Institute, St Thomas' Hospital, London, UK.

## Abstract

Hypoxia was assessed in three murine tumour models in vivo by measuring the incorporation of 7-(4'-(2-nitroimidazole-1-yl)-butyl)-theophylline (NITP), an immunologically identifiable hypoxia marker that binds bioreductively to cells under low-oxygen conditions. Proliferating cells were labelled in the same tumours by administering the thymidine analogue bromodeoxyuridine (BrdUrd). The relative hypoxia in each cell cycle phase of cells isolated from tumours was assessed by addition of propidium iodide with analysis by flow cytometry. There was no relationship between tumour volume and hypoxia in either the anaplastic sarcoma SaF or the poorly differentiated carcinoma CaNT and only a slight negative correlation in moderately well-differentiated carcinoma Rh. The G1/G0 phase contained the greatest number of aneuploid hypoxic cells (aneuploid hypoxia ranging from less than 1% up to 40%, 38% and 71% in SaF, CaNT and Rh respectively), although there were significant amounts of hypoxia present in S- and G2/M phases for all three tumours examined. However, the highest proportion of hypoxia occurred in the G2/M phase, in which up to 60% of the cells were hypoxic. Simultaneous measurement of hypoxia, proliferation and DNA content using a novel triple-staining flow cytometry method showed that hypoxic cells could actively participate in the cell cycle. In addition, the cell cycle distribution of NITP and BrdUrd labelling showed that hypoxic cells could progress through the cell cycle, although their rate of progression was slower than that of better oxygenated cells.


British Journal of Cancer (1998) 77(2), 227–234
© 1998 Cancer Research Campaign

### Cell cycle distribution of hypoxia and progression of hypoxic tumour cells in vivo

**L Webster[1], RJ Hodgkiss[2] and GD Wilson**

[1]Department of Immunology, The Rayne Institute, St Thomas' Hospital, Lambeth Palace Road, London SE1 7EH, UK; [2]Gray Laboratory Cancer Research Trust, PO Box 100, Mount Vernon Hospital, Northwood, Middlesex HA6 2JR, UK

**Summary** Hypoxia was assessed in three murine tumour models in vivo by measuring the incorporation of 7-(4′-(2-nitroimidazole-1-yl)-butyl)-theophylline (NITP), an immunologically identifiable hypoxia marker that binds bioreductively to cells under low-oxygen conditions. Proliferating cells were labelled in the same tumours by administering the thymidine analogue bromodeoxyuridine (BrdUrd). The relative hypoxia in each cell cycle phase of cells isolated from tumours was assessed by addition of propidium iodide with analysis by flow cytometry. There was no relationship between tumour volume and hypoxia in either the anaplastic sarcoma SaF or the poorly differentiated carcinoma CaNT and only a slight negative correlation in moderately well-differentiated carcinoma Rh. The $G_1/G_0$ phase contained the greatest number of aneuploid hypoxic cells (aneuploid hypoxia ranging from less than 1% up to 40%, 38% and 71% in SaF, CaNT and Rh respectively), although there were significant amounts of hypoxia present in S- and $G_2$/M phases for all three tumours examined. However, the highest proportion of hypoxia occurred in the $G_2$/M phase, in which up to 60% of the cells were hypoxic. Simultaneous measurement of hypoxia, proliferation and DNA content using a novel triple-staining flow cytometry method showed that hypoxic cells could actively participate in the cell cycle. In addition, the cell cycle distribution of NITP and BrdUrd labelling showed that hypoxic cells could progress through the cell cycle, although their rate of progression was slower than that of better oxygenated cells.

**Keywords:** proliferation; hypoxia; cell cycle; multi-parameter flow cytometry; tumour

Hypoxic radioresistant regions in some tumours are thought to reduce the efficacy of curative radiotherapy, and the sparing effect of proliferation of tumour cells during a course of conventional fractionated radiotherapy can be equivalent to several fractions of radiation. Proliferation can be studied by incorporation of the thymidine analogues bromo- and iododeoxyuridine, which can be identified using a monoclonal antibody with either flow cytometry analysis or immunohistochemical end points (Begg et al, 1985; Wilson et al, 1985). Measurements of potential doubling time (Tpot) are currently being undertaken in a series of trials comparing conventional with accelerated fractionation, and some show good evidence that pretreatment cell kinetics can predict treatment outcome (Wilson et al, 1988; Begg et al, 1992; Corvo et al, 1995). The degree of hypoxia within a solid tumour is important as hypoxic cells will be up to three times more resistant to radiotherapy than their better oxygenated counterparts (Gray et al, 1953). Meta-analysis of hypoxia-directed treatments has shown that regimens counteracting hypoxia in tumours are beneficial to patient survival, particularly in head and neck cancer (Overgaard, 1992). However, before further data can be obtained to support this conclusion, a reliable method of measuring clinical tumour hypoxia before treatment must be found. Several techniques are being developed and have progressed to clinical trial (Stone et al, 1993), with the Eppendorf oxygen microelectrode currently being the method of choice.

*Received 3 March 1997*
*Revised 18 June 1997*
*Accepted 7 July 1997*

*Correspondence to:* RJ Hodgkiss

Despite numerous studies of proliferation and hypoxia in tumours as individual factors affecting treatment outcome, there has been little investigation of these parameters in combination. The greater proportion of previous work investigated the cell cycle progress of cells in vitro subjected to extremes of hypoxia (Pettersen and Lindmo, 1983; Shrieve and Begg, 1985; Amellen and Pettersen, 1991), but few have attempted to study their interaction in vivo. Zeman et al (1993) found that in sequential histological sections of canine tumours there was no systematic relationship between hypoxia and proliferating cell nuclear antigen markers. However, clusters of proliferating cells were reported distal to blood vessels. In a small series of clinical soft-tissue sarcomas, Nordsmark et al (1996) reported that the fastest proliferating cells were found in the most hypoxic tumours (characterized by median $pO_2$), although there was no correlation with hypoxic fraction. Hypoxic sarcomas are also more likely to metastasize than better oxygenated tumours (Brizel et al, 1996). Hypoxic cells accumulate wild-type p53 (Graeber et al, 1994), which is required for efficient induction of apoptosis by hypoxia (Graeber et al, 1996). However, this mechanism also provides a selective pressure for outgrowth of cell lineages with mutated or deleted p53 in tumours, suggesting that hypoxia may ultimately select for a more aggressive phenotype.

We have measured hypoxia using bioreductive binding of NITP, which consists of a 2-nitroimidazole with an immunologically recognizable theophylline sidechain. The 2-nitroimidazole component binds to cellular macromolecules under low-oxygen conditions, and the bound adducts of the probe, in hypoxic cells, can then be identified and quantified by antibodies raised against the theophylline (Hodgkiss et al, 1991). S-phase tumour cells, actively synthesizing DNA in the cell cycle, can be labelled by their incorporation of the

**Figure 1** Typical flow cytometry profiles of cell nuclei prepared from SaF, CaNT and Rh murine tumours 2 h after treatment with NITP and BrdUrd in vivo and stained for hypoxia (bound NITP metabolites), proliferation (BrdUrd incorporation) and DNA content of cell nuclei, with simultaneous analysis of all three parameters in the SaF and CaNT tumours using three-colour flow cytometry. The regions on the SaF bivariate distributions represent the following cell populations: R1, hypoxic; R2, normoxic; R3, BrdUrd-labelled; R4, BrdUrd-unlabelled. Markers set on the SaF DNA histogram represent; M1, diploid host cells; M2, aneuploid tumour cells; M3, $G_1$; M4, S; M5, $G_2$/M

**Table 1** Summary of hypoxic fractions in SaF, CaNT and Rh murine tumours

| | Total hypoxia (%) | | Aneuploid hypoxia (%) | | Diploid hypoxia (%) | |
|---|---|---|---|---|---|---|
| Tumour | Mean | (range) | Mean | (range) | Mean | (range) |
| SaF | 9.9 | (< 1–29) | 11.6 | (< 1–40) | 5.5 | (< 1–15) |
| CaNT | 6.6 | (2–28) | 9.9 | (< 1–38) | 2.7 | (< 1–10) |
| Rh | 32.3 | (12–67) | 35.6 | (11–71) | 27.8 | (10–52) |

DNA precursor bromodeoxyuridine (BrdUrd). We have developed a novel three-colour staining flow cytometry method to measure the BrdUrd, NITP and DNA content of tumour cells, so that the interaction of these three parameters can be investigated (Webster et al, 1995). In this paper, we describe the cell cycle distribution of hypoxia in three murine tumour models by flow cytometry and follow the progress of hypoxic cells through the cell cycle by simultaneous evaluation of hypoxia (NITP), proliferation (BrdUrd) and DNA content.

### MATERIALS AND METHODS

### Mouse tumour models

The three tumours studied arose spontaneously in the Gray Laboratory animal colony (Hewitt and Wilson, 1961; Hewitt et al, 1973; Denekamp et al, 1980). The anaplastic sarcoma F (SaF) and the poorly differentiated mammary adenocarcinoma NT (CaNT) were transplanted in the syngeneic CBA/Ht Gy F TO mouse. The moderately well-differentiated Rhodesia adenocarcinoma (Rh) was transplanted in the syngeneic WHT/Gy F C57B1/10 mouse. Tumours were grown by inoculating subcutaneously a suspension of $10^5$ cells in 0.05 ml of 0.9% saline on the dorsum of mice. The tumours were selected for use, except when otherwise stated, at a mean diameter of 6 mm, determined using three orthogonal measurements with callipers. The latent periods for the SaF, CaNT and Rh tumours were approximately 10 days, 14 days and 6–10 weeks respectively.

### Hypoxia and proliferation marker administration

To label hypoxic cells, mice were injected i.p. with a formulation consisting of 0.04 M NITP dissolved in peanut oil with 10% dimethyl

© Cancer Research Campaign 1998

**Figure 2** The relationship between tumour volume and degree of aneuploid hypoxia in SaF, CaNT and Rh murine tumours labelled with NITP alone. Spearman's correlation coefficient (rho) for the dependence of aneuploid hypoxia on tumour volume was 0.139 ($P$ = 0.465), –0.0186 ($P$ = 0.925) and –0.385 ($P$ = 0.0432) respectively

**Figure 3** Cell cycle distribution of hypoxia within the aneuploid tumour cells for the three mouse tumour types (○, $G_1$; △, S; and ●, $G_2$/M) labelled with NITP alone. The data represent individual tumours arranged in ascending degree of hypoxia sulphoxide at a dose of 0.45 μmol g⁻¹ NITP (156 mg kg⁻¹, 0.38 ml per 35-g mouse). Proliferating cells were identified by administering 0.33 μmol g⁻¹ (100 mg kg⁻¹) BrdUrd i.p. at a concentration of 0.033 M in 0.9% saline. Animals were sacrificed 2 hours after NITP injection, tumours excised, weighed and minced finely with scissors. The tumour fragments were disaggregated into single-cell suspensions by enzyme digestion with 10 ml of 0.02% DNAase I, 0.2% collagenase IV (Sigma Chemical) in serum-free medium in a universal container on a rotating wheel for 30 min at 37°C. The cell suspensions were filtered through 35-μm nylon mesh and centrifuged for 10 min at 1000 r.p.m. Cell pellets were resuspended in 200 μl of phosphate buffered saline (PBS) and the single cells fixed by addition of 10 ml of 70% ethanol. The fixed cells were stored at least overnight at 4°C.

### Hypoxia staining for flow cytometry

The method has been reported previously (Hodgkiss et al, 1991). Briefly, $10^6$ ethanol-fixed cells were centrifuged in 5 ml of PBS at 2000 r.p.m. for 5 min and resuspended in neat rabbit antiserum to theophylline (Sigma, Dorset, UK) for 1 h. The cells were washed with 5 ml of PBS and resuspended in 0.25 ml of PBS containing 0.5%. Tween 20 and 0.1% normal goat serum (PNT) with 25 μl of IgG fluorescein isothiocyanate-conjugated goat anti-rabbit antiserum (Sigma) for 1 h. Cells were washed in 5 ml of PBS and the pellet resuspended in 2 ml of PBS containing 1 mg ml⁻¹ RNAase and 10 μg ml⁻¹ propidium iodide.

### Simultaneous measurement of hypoxia and proliferation by flow cytometry

The interdependence of cell cycle progression and oxygen status was studied by labelling hypoxic and proliferating cells according to two different time schedules. For the first schedule, SaF and CaNT tumours were labelled in vivo with BrdUrd at the start of the time course and then treated with NITP 2 h before each sacrifice time. For the second schedule, both NITP and BrdUrd were administered simultaneously and a range of sacrifice times used so that progression of labelled cells through the cell cycle could be observed.

The method for simultaneous measurement of hypoxia proliferation and DNA content has been reported previously (Webster et al, 1995). Briefly, $10^6$ ethanol-fixed cells were washed in PBS, centrifuged for 5 min at 2000 r.p.m. and the pellet resuspended in 0.2 mg ml⁻¹ pepsin in 2 M hydrochloric acid for 20 min at room temperature. The resultant nuclei were washed twice in PBS and re-suspended in 100 μl of PNT containing 5 μl of mouse monoclonal to BrdUrd (Dako, Bucks, UK). After 1 h incubation, the nuclei were washed and the pellet resuspended in 100 μl of PNT with 25 μl of R-phycoerythrin-conjugated anti-mouse lgG (Dako) for a further hour. After washing, the pellet was resuspended in 250 μl of undiluted rabbit anti-theophylline antiserum for 1 h. The final antibody solution, 250 μl of PNT with 25 μl of anti-rabbit IgG FITC conjugate, was added after a further wash and also incubated

© Cancer Research Campaign 1998

**Table 2** Analysis of cell cycle distribution of hypoxia (Figure 3) for SaF, CaNT and Rh murine tumours

| | Regression analysis | | Paired *t*-test | |
|---|---|---|---|---|
| Tumour | Comparison | Slope ± s.e.m. | Mean difference | *P (t)* |
| SaF | $G_1$ vs S | 0.432 ± 0.018 | 3.908 | < 0.0001 |
| | $G_1$ vs $G_2$ | 0.260 ± 0.021 | 4.827 | < 0.0001 |
| | $G_2$ vs S | 0.604 ± 0.037 | 0.919 | < 0.0001 |
| CaNT | $G_1$ vs S | 0.216 ± 0.022 | 4.431 | < 0.0001 |
| | $G_1$ vs $G_2$ | 0.241 ± 0.019 | 3.851 | < 0.0001 |
| | $G_2$ vs S | 0.881 ± 0.105 | –0.580 | < 0.0005 |
| Rh | $G_1$ vs S | 4.198 ± 0.769 | 18.106 | < 0.0001 |
| | $G_1$ vs $G_2$ | 0.143 ± 0.020 | 17.624 | < 0.0001 |
| | $G_2$ vs S | 0.779 ± 0.149 | –0.482 | 0.1781 |

**Table 3** Analysis of cell cycle phase-specific hypoxia (Figure 4) for SaF, CaNT and Rh murine tumours

| | Regression analysis | | Paired *t*-test | |
|---|---|---|---|---|
| Tumour | Comparison | Slope ± s.e.m. | Mean difference | *P (t)* |
| SaF | S vs $G_1$ | 1.232 ± 0.110 | –2.365 | < 0.0001 |
| | $G_2$ vs $G_1$ | 0.315 ± 0.036 | 13.570 | < 0.0001 |
| | $G_2$ vs S | 0.215 ± 0.030 | 15.935 | < 0.0001 |
| CaNT | S vs $G_1$ | 0.653 ± 0.057 | 2.660 | < 0.0038 |
| | $G_2$ vs $G_1$ | 0.450 ± 0.038 | 9.604 | < 0.0001 |
| | $G_2$ vs S | 0.668 ± 0.029 | 6.944 | < 0.0001 |
| Rh | S vs $G_1$ | 0.622 ± 0.130 | 3.497 | 0.0433 |
| | $G_2$ vs $G_1$ | 0.625 ± 0.063 | 16.399 | < 0.0001 |
| | $G_2$ vs S | 0.511 ± 0.110 | 12.902 | < 0.0001 |

**Figure 4** Cell cycle phase-specific hypoxia within the aneuploid tumour cells for the three mouse tumour types (O, $G_1$; Δ, S; and ●, $G_2$/M) labelled with NITP alone. The data represent individual tumours arranged in ascending degree of hypoxia for 1 h. Nuclei were washed for the final time and resuspended in 2 ml of PBS with 20 μl of 1 mg $ml^{-1}$ 7-aminoactinomycin D solution (Sigma).

### Flow cytometry

All samples were run on a Becton Dickinson FACScan with a single excitation wavelength of 488 nm. Red fluorescence from either propidium iodide or 7-aminoactinomycin D bound to the DNA was used to select single cells and reject debris and cell clumps on the basis of their DNA content using the doublet discrimination mode. Light scatter and fluorescence data were collected for $10^4$ single cells, with bound NITP (hypoxia) represented by green fluorescence (FITC), incorporated BrdUrd (proliferation) by orange/red fluorescence (phycoerythrin) and DNA by red fluorescence (either propidium iodide or 7-aminoactinomycin D). Compensation was applied for the triple-stained samples to allow for overlap in the fluorochrome emission spectra and enable the orange/red fluorescence (phycoerythrin) baseline to approach a horizontal position.

### Statistical analysis

The dependence of hypoxic fraction on tumour volume was assessed by Spearman's correlation analysis using the JMP statistical package, SAS Institute. The cell cycle phase-dependence of hypoxia was analysed by regression analysis with a paired *t*-test comparison of data in the different cell cycle phases, also using JMP.

### RESULTS

### Hypoxia related to tumour size

Tumours were selected with geometric mean diameters of 4–12 mm for assessment of hypoxia. The tumours were analysed using a DNA histogram and a bivariate distribution of bound NITP (hypoxia) vs DNA content. Typical profiles for SaF, CaNT and Rh tumours are displayed in Figure 1, with the appropriate analysis regions indicated. The hypoxic (R1) and non-hypoxic (R2) regions were defined using the non-specific background staining from an appropriate control tumour not treated with NITP, but excised and taken through the staining procedure. All three tumour types exhibited both diploid and aneuploid cell populations, and in these experiments the flow cytometer settings were adjusted so that the aneuploid tumour cells were always in approximately the same position on the DNA baseline. The position of the diploid $G_1$ peak on DNA histograms is identical to that of the diploid $G_1$ cells from murine bone marrow (data not shown). Based on studies of cell morphology, the predominant normal cell types were lymphocytes and macrophages (SA Hill, personal communication). When cultured in vitro, the diploid cells from both SaF and CaNT tumours fail to proliferate and only

© Cancer Research Campaign 1998

**Figure 5** Progression of BrdUrd-labelled cells in murine tumours after labelling with BrdUrd. NITP was administered 2 h before sacrifice. The proportion of aneuploid BrdUrd labelling in (**A**) CaNT $G_1$ phase, (**B**) CaNT $G_2$ phase, (**C**) SaF $G_1$ phase and (**D**) SaF $G_2$ phase. ●, Oxic cells expressed as oxic BrdUrd-labelled cells in phase/total oxic BrdUrd-labelled cells; ○, hypoxic cells expressed as hypoxic BrdUrd-labelled cells in phase / total hypoxic BrdUrd-labelled cells the aneuploid cells are represented in the culture after a few days (data not shown).

Using the regions shown in Figure 1, total hypoxia (R1) including both diploid and aneuploid cells can be calculated. Diploid and aneuploid hypoxia were estimated from a DNA histogram gated on R1, with markers (M1 and M2 respectively) set for the appropriate populations. The results for total, aneuploid and diploid hypoxia for SaF, CaNT and Rh tumours are shown in Table 1. There is a wide inter- and intra-tumour variation in aneuploid hypoxia with all three tumour types. The diploid population of cells, consisting of macrophages, lymphocytes and stromal cells, also showed hypoxia but to a lesser extent than the aneuploid population, and the mean fluorescence of the diploid population was lower than that of the aneuploid cells. This was especially noticeable in the diploid $G_2/M$ population amid the aneuploid S-phase cells of the Rh tumour (Figure 1).

Figure 2 shows the relationship between aneuploid hypoxia and tumour volume. At these macroscopic sizes, there was no correlation between volume and hypoxia in the Saf and CaNT tumours (Spearman's correlation coefficient rho = 0.139, $P$ = 0.465 and –0.0186, $P$ = 0.925), respectively, while the Rh tumour showed a slight trend that was just significant (rho = –0.385, $P$ = 0.0432) for hypoxia to fall with increasing volume.

### Cell cycle distribution of hypoxia

Analysis of the distribution of hypoxia within the cell cycle was carried out by setting markers (M2 to M5) on DNA histograms gated on the hypoxic and oxic cells (Figure 1). Hypoxia was present throughout the cell cycle (Figure 3 and Table 2), and the $G_1/G_0$ phase contained the greatest number of hypoxic cells and was significantly different from the S- and $G_2$ phases (Table 2). In

© Cancer Research Campaign 1998

**Figure 6** Progression of BrdUrd-labelled cells in SaF tumours after simultaneous labelling with NITP and BrdUrd. The proportion of aneuploid BrdUrd labelling in (**A**) $G_1$ phase, (**B**) $G_2$ phase. ●, Oxic cells expressed as oxic BrdUrd-labelled cells in phase/total oxic BrdUrd-labelled cells; ○, hypoxic cells expressed as hypoxic BrdUrd-labelled cells in phase/total hypoxic BrdUrd-labelled cells the SaF $G_1/G_0$ hypoxia ranged from < 1% to 24% in the CaNT from < 1% to 23% and in the Rh from < 1% to 58% of the aneuploid hypoxic population. This is not surprising as, in all three tumours, $G_1/G_0$ represents 40% of the total cell population. In all three tumour types, when hypoxia was present, S- and $G_2$/M phases also contained significant numbers of hypoxic cells.

However, calculation of the proportion of hypoxic cells within each individual phase of the cell cycle showed that, although the greatest number of hypoxic cells resided in $G_1/G_0$, the phase of the cell cycle with the highest proportion of hypoxia was $G_2$/M (Figure 4 and Table 3), with typically a two-fold lower proportion of hypoxic cells within the $G_0/G_1$ and S-phases. This effect was most marked in the SaF tumour, but similar patterns were also seen in the CaNT and Rh tumours, and in each case $G_2$ was significantly different from the $G_1$ and S-phases (Table 3).

### Cell progression studies

Analysis of cell progression in CaNT and SaF tumours labelled with NITP and BrdUrd was carried out using the regions illustrated in Figure 1, set on bivariate distributions of hypoxia or proliferation vs DNA content. From these, gated DNA histograms were created to include only cells labelled with either both BrdUrd and NITP (R1 and R3) or cells labelled with BrdUrd and not with NITP (R2 and R3). Markers (M2 to M5) set on these DNA histograms allowed the number of cells within each phase of the aneuploid cell cycle to be calculated for both labelled subgroups. The stromal host cells were not included in this analysis, and previous work has shown that the majority of these cells show little progression.

Figure 5 shows the time dependence of BrdUrd labelling in the $G_1$ and $G_2$ phases of the cell cycle in hypoxic and normoxic aneuploid cells from CaNT and SaF solid tumours that were labelled in vivo with BrdUrd at the start of the experiment and with NITP during the 2-h period before each time point. Movement of BrdUrd-labelled cells through mitosis into $G_1$ occurs more rapidly in the normoxic population than in the hypoxic cells (Figures 5A and C) in both tumour types, and there is a corresponding delay in progression of labelled cells out of $G_2$ in hypoxic compared with normoxic cells, although this delay is more obvious in CaNT tumours (Figure 5B) than in SaF tumours (Figure 5D). In both tumour types, 10–20% of both hypoxic and normoxic BrdUrd-labelled cells that enter $G_2$ show little cell cycle progression over the 30-h time course of the experiment.

Figure 6 shows the time dependence of BrdUrd labelling in SaF tumours, in which both proliferating S-phase cells and hypoxic cells were labelled with both BrdUrd and NITP, respectively, at the start of the experiment. Both incorporated BrdUrd and bound NITP could be reliably identified in cells from tumours 28 h after administration, and the pattern of progression of labelled cells through the cell cycle was broadly similar to that seen with staggered proliferation and hypoxia marker administration. The BrdUrd-labelled cells within each phase of the cell cycle were classified as hypoxic (NITP binding) or oxic (no NITP binding). Each cell cycle phase was expressed as a percentage of the total BrdUrd-labelled cells so that the values were unaffected by progression of unlabelled cells. The time course of progression of BrdUrd-labelled cells through all three phases of the cell cycle was similar. However, progression of hypoxic cells through mitosis into $G_1$ was slightly delayed compared with that of normoxic cells (Figure 6A), and this was probably related to a transient build-up of hypoxic cells in $G_2$ (Figure 6B) before passing through mitosis. A proportion (10%) of BrdUrd-labelled cells appear to arrest permanently in $G_2$ rather than progressing through mitosis.

### DISCUSSION

Metabolic binding of NITP has a similar dependency on oxygen concentration to that of radiosensitivity (Hodgkiss et al, 1991). The $K$-value for binding (1400 p.p.m. oxygen) compared with the $K$-value (3800 p.p.m. oxygen) for radiosensitivity under identical conditions suggests that NITP binding requires slightly more stringent hypoxia than that needed for radioresistance. The majority of the NITP binding observed is therefore to relatively hypoxic radioresistant cells at oxygen tensions below the $K$-value for radiosensitivity. Tumours contain gradients of oxygen (and other nutrients) and the concept of hypoxic and well-oxygenated populations is an oversimplification. The population defined as hypoxic is probably heterogeneous in both the level and duration of low oxygen tensions to which the individual cells have been subjected. Some of the NITP binding may reflect different stringencies of hypoxia or, alternatively, may reflect acute variations in oxygen tension during the period of drug binding; it is not possible to distinguish between these alternatives at present.

© Cancer Research Campaign 1998

### Tumour size

The hypoxic fraction is thought to consistently increase with tumour size as the tumour grows from a microscopic to a macroscopic lesion, in which some cells are further from a blood capillary than the diffusion distance of oxygen through tissues. Within the macroscopic range, the relationship of tumour size to hypoxic fraction is less clear (Moulder and Rockwell, 1984). However, when some specific tumours were compared at different sizes, there was a trend for the hypoxic fraction to increase as size increased, although this was not true for all tumour types (Stanley et al, 1977; Siemann, 1980; Wallen et al, 1980; Okunieff et al, 1986; Fu et al, 1990). Over the size range of 4- to 12-mm diameter, SaF, CaNT and Rh tumours showed no correlation between size and hypoxic fraction measured by flow cytometric analysis of bioreductively bound NITP abducts (Figure 2). The range in hypoxic fraction was large, even for tumours of similar diameter or weight. Inter-tumoral differences in hypoxic binding are not related to differences in drug (NITP) delivery (Hodgkiss et al, 1995) or to poor drug distribution, as immunostaining of sections of large tumours (10-mm diameter) shows hypoxia to be distributed throughout the tumour section, although with a variable pattern depending on the structure of the tumour (unpublished data). The three tumours selected show a corded pattern (CaNT; Hodgkiss et al, 1991), a patchy distribution (SaF) and a random, highly hypoxic pattern (Rh). The estimation by flow cytometry of mean hypoxic fractions of 11.6%, 12.9% and 35.6% for the SaF, CaNT and Rh tumours, respectively, is supported by the greater density and more widespread staining in sections of the Rh tumour compared with sections of the other tumours.

### Hypoxia and cell cycle

Profiles of hypoxic cells produced by NITP and flow cytometry showed that most hypoxic cells were in the $G_1/G_0$ population, although hypoxia occurred throughout the cell cycle (Figure 3 and Table 2). Most of the cells in tumours tend to be in $G_1/G_0$ and the predominance of $G_1$ cells in the hypoxic subpopulation probably reflects this. Cells are thought to arrest in $G_1$ as they move away from blood vessels, becoming hypoxic and nutritionally deprived (Tannock, 1968; Hirst and Denekamp, 1979), and finally enter the quiescent $G_0$ phase. Similar observations have been made in spheroids in which the proportion of nutritionally deprived cells and the percentage of $G_1$ cells increases with size, while the proportion of those in the S- and $G_2$ phases decreases (Sutherland et al, 1986). However, our observation of significant numbers of hypoxic S and $G_2$/M cells is also supported by other investigators (Pallavicini et al, 1979; Siemann and Keng, 1988).

Within each cell cycle phase (defined by DNA content) in SaF, CaNT and Rh tumours, the highest proportion of hypoxic cells was located within $G_2$ (Figure 4 and Table 3). Most in vitro cell cycle studies have shown that induction of hypoxia produces either total arrest of the cell cycle or cell cycle-specific arrest (Pettersen and Lindmo, 1983; Shrieve and Begg, 1985; Amellem and Pettersen, 1991). Survival studies on different components of the cell cycle have shown S-phase to be the most sensitive to hypoxic treatment, with sensitivity slightly reduced in $G_1$ and greatly reduced in $G_2$ (Spiro et al, 1984). Entry of cells into S is often blocked with an accumulation at the $G_1$/S border and cells can be either totally arrested in S such that no further DNA synthesis occurs, or progress slowly through S-phase with synthesis of DNA over a prolonged period (Pettersen and Lindmo, 1983). Cells in $G_2$/M have been reported to be insensitive to hypoxia, progressing through mitosis to $G_1$, where arrest may occur. In the in vivo studies presented here, a high proportion of hypoxic cells in $G_2$ may have completed a hypoxic S-phase, perhaps with a reduced fidelity of DNA synthesis, and then progress slowly or are unable to continue through mitosis. Wilson et al (1994) reported that 20–25% of cells in untreated BrdUrd-labelled cells in SaF and Rh tumours arrested in $G_2$ in the first cycle. Some of these $G_2$ cells may no longer be clonogenic and may be committed to die from signals related to hypoxia or nutritional status.

### Measurement of proliferation and hypoxia

Following BrdUrd-labelled cells through the cell cycle and assessing their hypoxia at time intervals showed that cells actively progressing through the cell cycle could become hypoxic (Figure 5) and that their progression was delayed relative to the better oxygenated component. The hypoxic status of cells can only be determined at the time of labelling, and reoxygenation at other times can not be excluded and may account for some of the cell cycle progression. Slower progression of hypoxic compared with normoxic cells through $G_2$ leads to a temporary accumulation of hypoxic $G_2$ cells in CaNT tumours (Figure 5B) and delays their entry into $G_1$ (Figure 5A). Similar but less marked trends are also observed in SaF tumours, in which progression of hypoxic cells appears to be relatively slow. In both tumour types a proportion of $G_2$ cells became permanently arrested, and this appeared to be more closely related to oxygenation status in CaNT rather than in SaF tumours, in which other factors may be more important. It may be hypothesized that progression of the hypoxic cells in both tumour types is delayed because of deprivation of oxygen or other nutrients, but in these experiments chronically and acutely hypoxic cells can not be distinguished.

Labelling SaF tumours simultaneously with proliferation (BrdUrd) and hypoxia (NITP) markers and following the progress of this BrdUrd-labelled cohort through the cell cycle supported these observations (Figure 6). The presence of significant NITP binding 28 h after labelling demonstrated persistence of hypoxic cells in these tumours. Some progression through the cell cycle of these hypoxic S-phase cells was observed during this time. Slower progression of hypoxic compared with normoxic cells through $G_2$ led to a temporary accumulation of hypoxic $G_2$ cells 8 h after labelling (Figure 6B) and delayed their entry into $G_1$ (Figure 6A). The observed delay in entry of hypoxic compared with oxic cells into $G_1$ was much shorter after simultaneous compared with staggered administration of proliferation and hypoxia markers and may be evidence for reoxygenation of part of the hypoxic population during the time course of the experiment.

The $G_2$ block seen after both staggered and simultaneous labelling with hypoxia and proliferation markers could reflect hypoxic S-phase DNA synthesis, which may increase the probability of faulty replication of DNA. p53 is induced by hypoxia and DNA damage and is responsible for cell cycle arrest in both $G_1$ (Cox and Lane, 1995; Shimamura and Fisher, 1996) and $G_2$ (Stewart et al, 1995). A $G_2$ delay would allow post-replication repair to occur, with sectoring of genetically damaged cells to undergo apoptosis or necrosis. A $G_2$ block may also create a reservoir of pre-mitotic cells. In vitro modelling has shown that cells do not tend to enter DNA synthesis unless they have a good nutrient and oxygen supply. Gelfant (1977) also showed in vivo that mouse

© Cancer Research Campaign 1998 

epithelium contained a number of cells permanently arrested in $G_2$ that remained there for at least several months. These cells were activated when the tissue was injured to immediately repopulate the area through mitosis, while other cells initiated DNA synthesis. A reservoir of $G_2$ cells in unfavourable (hypoxic) conditions could allow rapid repopulation if more favourable conditions within the tumour were restored.

In this study of cell cycle progression and hypoxia in vivo, evidence has been presented to show that hypoxic cells in tumours can not only actively synthesize DNA, but that some cell cycle progression is possible under hypoxic conditions, although at a slower rate than in better oxygenated cells. Hypoxic S-phase cells exhibit delayed progression through $G_2$ into mitosis, and this may indicate a cell cycle checkpoint in $G_2$ at which the fidelity of DNA synthesis in hypoxic or nutrient-deprived environments is monitored or DNA repair carried out. These studies demonstrate an approach towards elucidating the complex interactions between the hypoxic and proliferating compartments in tumours, which will help to identify the mechanisms involved in cell cycle regulation.

### ACKNOWLEDGEMENTS

This work was supported by the Cancer Research Campaign.

### REFERENCES

Amellem O and Pettersen EO (1991) Cell inactivation and cell cycle inhibition as induced by extreme hypoxia: the possible role of cell cycle arrest as a protection against hypoxia-induced lethal damage. *Cell Prolif* **24**: 127–141

Begg AC, McNally NJ, Shrieve DC and Karcher H (1985) A method to measure the DNA synthesis and the potential doubling time from a single sample. *Cytometry* **6**: 620–626

Begg AC, Hofland I, van Glabekke M, Bartelink H and Horiot JC (1992) Predictive value of potential doubling time for radiotherapy of head and neck tumour patients: results from the EORTC co-operative trial 22851. *Semin Rad Oncol* **2**: 22–25

Brizel DM, Scully SP, Harrelson JM, Layfield LJ, Bean JM, Prosnitz LR and Dewhirst MW (1996) Tumour oxygenation predicts for the likelihood of distant metastases in human soft tissue sarcoma. *Cancer Res* **56**: 941–943

Corvo R, Giaretti W, Sanguineti G, Geido E, Orecchia R, Guenzi M, Margarino G, Bacigalupo A, Garaventa G, Barbieri M and Vitale V (1995) In vivo cell kinetics in head and neck squamous cell carcinomas predicts local control and helps guide radiotherapy regimen. *J Clin Oncol* **13**: 1843–1850

Cox LS and Lane DP (1995) Tumour suppressors, kinases and clamps: how p53 regulates the cell cycle in response to DNA damage. *Bioessays* **17**: 501–508

Denekamp J, Hirst DG, Stewart FA and Terry NH (1980) Is tumour radiosensitization by misonidazole a general phenomenon? *Br J Cancer* **41**: 1–9

Fu KK, Wendland MF, Iyer SB, Lam KN, Engeseth H and James TL (1990) Correlations between in vivo $^{31}$P NMR spectroscopy measurements, tumour size, hypoxic fraction and cell survival after radiotherapy. *Int J Rad Oncol Biol Phys* **18**: 1341–1350

Gelfant S (1977) A new concept of tissue and tumour cell proliferation. *Cancer Res* **37**: 3845–3862

Graeber TG, Peterson JF, Tsai M, Monica K, Fornace AJ Jr and Giaccia AJ (1994) Hypoxia induces accumulation of p53 protein, but activation of a G1-phase checkpoint by low-oxygen conditions is independent of p53 status. *Mol Cell Biol* **14**: 6264–6277

Graeber TG, Osmanian C, Jacks T, Housman DE, Koch CJ, Lowe SW and Giaccia AJ (1996) Hypoxia-mediated selection of cells with diminished apoptotic potential in solid tumours (see comments). *Nature* **379**: 88–91

Gray LH, Conger AD, Ebert M, Hornsey S and Scott OCA (1953) The concentration of oxygen dissolved in tissues at the time of irradiation as a factor in radiotherapy. *Br J Radiol* **26**: 638–648

Hewitt HB and Wilson CW (1961) Survival curves for tumour cells irradiated in vitro. *Ann NY Acad Sci* **95**: 818–827

Hewitt HB, Blake E and Porter EH (1973) The effect of lethally irradiated cells on the transplantability of murine tumours. *Br J Cancer* **28**: 123–135

Hirst DG and Denekamp J (1979) Tumour cell proliferation in relation to the vasculature. *Cell Tissue Kinet* **12**: 31–42

Hodgkiss RJ, Jones G, Long A, Parrick J, Smith KA, Stratford MRL and Wilson GD (1991) Flow cytometric evaluation of hypoxic cells in solid experimental tumours using fluorescence immunodetection. *Br J Cancer* **63**: 119–125

Hodgkiss RJ, Stratford MRL, Dennis MF and Hill SA (1995) Pharmacokinetics and binding of the bioreductive probe for hypoxia, NITP: effect of route of administration. *Br J Cancer* **72**: 1462–1468

Moulder JE and Rockwell S (1984) Hypoxic fractions of solid tumours: experimental techniques, methods of analysis and a survey of existing data. *Int J Rad Oncol Biol Phys* **10**: 695–712

Nordsmark M, Hoyer M, Keller J, Nielsen OS, Jensen OM and Overgaard J (1996) *Int J Rad Oncol Biol Phys* **35**: 701–708

Okunieff PG, Neuringer L and Suit HD (1986) Tumour size dependent changes in a murine fibrosarcoma: use of in vivo $^{31}$P NMR for non-invasive evaluation of tumour metabolic status. *Int J Rad Oncol Biol Phys* **12**: 793–79

Overgaard J (1992) Importance of tumour hypoxia in radiotherapy: a meta-analysis of controlled clinical trials. *Radiother Oncol* **24**: S64

Pallavicini MG, Lalande ME, Miller RG and Hill RP (1979) Cell cycle distribution of chronically hypoxic cells and determination of the clonogenic potential of cells accumulated in G2+M phases after irradiation of a solid tumour in vivo. *Cancer Res* **39**: 1891–1897

Pettersen EO and Lindmo T (1983) Inhibition of cell cycle progression by acute treatment with various degrees of hypoxia: modifications induced by low concentrations of misonidazole present during hypoxia. *Br J Cancer* **48**: 809–817

Shimamura A and Fisher DE (1996) p53 in life and death. *Clin Cancer Res* **2**: 435–440

Shrieve DC and Begg AC (1985) Cell cycle kinetics of aerated, hypoxic and re-aerated cells in vitro using flow cytometric determination of cellular DNA and incorporated bromodeoxyuridine. *Cell Tissue Kinet* **18**: 641–651

Siemann DW (1980) Tumour size: a factor influencing the isoeffect analysis of tumour response to combined modalities. *Br J Cancer* **42** (suppl. 4): 294–298

Siemann DW and Keng PC (1988) Characterisation of radiation resistant hypoxic cell subpopulations in KHT sarcomas. II. Cell sorting. *Br J Cancer* **58**: 296–300

Spiro IJ, Rice GC, Durand RE, Stickler R and Ling CC (1984) Cell killing, radiosensitization and cell cycle redistribution induced by chronic hypoxia. *Int J Rad Oncol Biol Phys* **10**: 1275–1280

Stanely JA, Shipley WU and Steel GG (1977) Influence of tumour size on hypoxic fraction and therapeutic sensitivity of Lewis lung tumour. *Br J Cancer* **36**: 105–113

Stewart N, Hicks GG, Paraskevas F and Mowat M (1995) Evidence for a second cell cycle block at G2/M by p53. *Oncogene* **10**: 109–116

Stone HB, Brown JM, Phillips TL and Sutherland RM (1993) Oxygen in human tumours: correlations between methods of measurement and response to therapy. *Rad Res* **136**: 422–434

Sutherland RM, Sordat B, Bamat J, Gabbert H, Bourrat B and Mueller-Klieser W (1986) Oxygenation and differentiation in multicellular spheroids of human colon carcinoma. *Cancer Res* **46**: 5320–5329

Tannock IF (1968) The relation between cell proliferation and the vascular system in a transplanted mouse mammary tumour. *Br J Cancer* **22**: 258–273

Wallen CA, Michaelson SM and Wheeler KT (1980) Evidence for an unconventional radiosensitivity of rat 9L subcutaneous tumours. *Rad Res* **84**: 529–541

Webster L, Hodgkiss RJ and Wilson GD (1995) Simultaneous triple staining for hypoxia, proliferation and DNA content in solid murine tumours. *Cytometry* **21**: 344–351

Wilson GD, McNally NJ, Dische S, Saunders MI, Des Rochers C, Lewis AA and Bennet MH (1988) Measurement of cell kinetics in human tumours in vivo using bromodeoxyuridine incorporation and flow cytometry. *Br J Cancer* **58**: 423–431

Wilson GD, McNally NJ, Dunphy E, Karcher H and Pfragner R (1985) The labelling index of human and mouse tumours assessed by bromodeoxyuridine staining in vitro and in vivo and flow cytometry. *Cytometry* **6**: 641–647

Wilson GD, Martindale CA, Soranson JA, Bourhis J, Carl UM and McNally NJ (1994) Radiation-induced cell cycle delay measured in two mouse tumours in vivo using bromodeoxyuridine. *Rad Res* **137**: 177–185

Zeman EM, Calkins DP, Cline JM, Thrall DE and Raleigh JA (1993) The relationship between proliferative and oxygenation status in spontaneous canine tumours. *Int J Radiat Oncol Biol Phys* **27**: 891–898

© Cancer Research Campaign 1998